# Healthcare workers’ priorities of WHO snakebite strategic objectives for the control and prevention of snakebite envenoming in Ghana: A machine learning statistical design of experiment modeling

**DOI:** 10.1371/journal.pntd.0013295

**Published:** 2025-07-10

**Authors:** Eric Nyarko, Iddrisu Abugbil Atubiga, Emmanuel Tetteh Siame, José María Gutiérrez, Eduardo Alberto Fernandez

**Affiliations:** 1 Department of Statistics and Actuarial Science, School of Physical and Mathematical Sciences, College of Basic and Applied Sciences, University of Ghana, Legon, Accra, Ghana; 2 Instituto Clodomiro Picado, Facultad de Microbiología, Universidad de Costa Rica, San José, Costa Rica; 3 Department of Health Sciences, Brock University, St Catharines, Ontario, Canada; Fundação de Medicina Tropical Doutor Heitor Vieira Dourado: Fundacao de Medicina Tropical Doutor Heitor Vieira Dourado, BRAZIL

## Abstract

**Background:**

Snakebite is a severe neglected tropical disease (NTD) that affects 2.5 million people each year, resulting in the deaths of 81,000–138,000 individuals, including rural villagers, agricultural workers, and children. The World Health Organization (WHO) has set strategic objectives to halve the deaths and disabilities caused by snakebite envenoming (SBE) by 2030. This study used innovative research methods, such as the statistical design of experiments and machine learning (ML), to explore healthcare workers’ priorities in Ghana regarding the WHO’s strategic objectives for controlling and preventing SBE. The goal was to identify their priority needs to guide the development of a research agenda and relevant interventions or policies that prioritize local needs while aligning with the WHO’s strategic objectives for SBE control and prevention.

**Method:**

In this cross-sectional study, we employed a MaxDiff statistical design to collect data on the prioritization of the WHO strategic objectives for SBE from 137 healthcare workers in the Kwahu Afram Plains North and South districts of the Eastern Region of Ghana from August to December 2024. We divided the final dataset using a hold-back validation method, maintaining a training-to-validation ratio of 70:30. For data analysis, we utilized a diverse range of five machine learning models: Ridge Regression, Elastic Net, LASSO, a Generalized Regression Model with Pruned Forward Selection, and Forward Selection. To compare the performance of these models, we used several key metrics, including Akaike Information Criterion corrected (AICc), the Bayesian Information Criterion (BIC), the Root Average Squared Error (RASE), negative log-likelihood, and the total time taken to fit each model.

**Results:**

The Ridge regression model appeared as the best candidate among the ML models used in this study. Its superior predictive performance justifies the computational cost it requires, making it the preferred option for applications that prioritize both predictive performance and computational efficiency. This model consistently predicted key WHO strategic objectives for preventing and controlling SBE. Of the objectives, ‘Ensuring safe and effective treatment’ had the highest priority, followed by ‘Strengthening health systems’, ‘Empowering and engaging communities’ and ‘Increasing partnerships, coordination, and resources’. This underscores their order of importance for local initiatives. Therefore, these strategies must be prioritized when designing local policies, relevant interventions, and research agendas.

**Conclusion:**

By utilizing a MaxDiff statistical experiment design and five machine learning models, participants prioritized the WHO strategic objectives for preventing and controlling SBE in Ghana. Our findings provide essential insights into local policy-making and intervention strategies and for shaping research agendas in Ghana. A local action plan is urgently needed, prioritizing ‘Ensuring safe and effective treatment’ at the community level, followed by ‘Strengthening health systems’, ‘Empowering and engaging communities’, and ‘Increasing partnerships, coordination, and resources’. Prioritizing these strategies in Ghana is crucial for supporting the WHO’s goal of reducing the global SBE burden by 50% by 2030. The success of these strategies hinges on the active involvement of the Ministry of Health and the Ghana Health Service in their implementation at the local level and within the health system.

## Author summary

The WHO has set ambitious strategic objectives to halve the deaths and disabilities caused by SBE by 2030. However, significant challenges exist in sub-Saharan Africa, including a lack of rigorous research evidence and a misalignment of research with local priorities. Comprehensive research is essential to identifying country-level priorities to guide the design and implementation of locally relevant plans for combating this NTD. Achieving this goal demands innovative research methods, including expanding the traditional health research toolkit by adapting existing methodologies and incorporating new approaches to gather improved information on snakebites in Ghana and the sub-region. To this end, this study employed innovative research methods, including the statistical design of experiments and machine learning models, to identify healthcare workers’ priorities regarding the WHO’s strategic objectives for controlling and preventing SBE. Our findings indicate that healthcare workers in a particular region of Ghana prioritized ‘Ensuring safe and effective treatment’, followed by ‘Strengthening health systems’, ‘Empowering and engaging communities’, and ‘Increasing partnerships, coordination, and resources’. Implementing a local action plan, as our research suggests, has the potential to significantly impact the health landscape, offering hope for communities at risk of snakebite and snakebite victims. Prioritizing these strategies in Ghana is crucial for supporting the WHO’s goal of reducing the global SBE burden by 50% by 2030. However, the success of these strategies hinges on the active involvement of the Ministry of Health and the Ghana Health Service in their implementation at the local level and within the health system.

## Introduction

Snakebite envenoming (SBE) is an urgent public health issue, claiming the lives of 81,000–138,000 people annually worldwide. It disproportionately affects rural villagers, agricultural workers, women, young males, and children, leaving around 400,000 individuals disabled. The most severe impact is observed in tropical and subtropical regions of Africa, Asia, and Latin America [1–5]. Hospital-based data from sub-Saharan Africa (SSA) indicates an annual incidence of snakebites of 56.4 per 100,000 people, with a mortality rate of 1.35 per 100,000 per year in rural areas [2]. However, these data may only represent a portion of the snakebite burden in Africa, primarily from specific regions known for high snakebite rates [6].

In 2017, SBE was classified as a Category A neglected tropical disease (NTD) by the WHO [7], which was followed by a resolution from the World Health Assembly in 2018 [8]. In 2019, the WHO launched a global strategy for the prevention and control of SBE, aiming to reduce deaths and disabilities caused by SBE by 50% and to provide three million effective treatments annually by 2030 [9]. The strategy includes four ambitious objectives: (a) Ensuring safe, effective treatment, (b) Strengthening health systems, (c) Empowering and engaging communities, and (d) Increasing partnerships, coordination, and resources. Since the launch of this strategy, there has been growing interest from decision-makers for high-quality research evidence to inform suitable policies and interventions [10,11], including the design and implementation of locally relevant plans [12]. During the full rollout of the strategy from 2025 to 2030, all countries are expected to integrate it into their national public health agendas, with the WHO planning to review and adapt the strategy by 2030 to meet the specific needs of the countries most affected [12]. Therefore, it is crucial to identify country-level priorities through rigorous research evidence to guide the design and implementation of locally relevant plans for combating this NTD. Achieving this goal will require innovative and advanced research methods. This means expanding the traditional health research toolkit by adapting existing methodologies and incorporating new tools to gather better information on snakebites in Ghana [13]. Such efforts will provide a stronger foundation for identifying community-level priorities that inform the development of a research agenda and relevant interventions/policies meeting local needs.

Artificial intelligence (AI), including machine learning (ML) and deep learning, holds immense promise for contributing to this effort. With their innovative approach and potentially high predictive power, AI/ML algorithms offer a new frontier in data analysis [14]. While existing snakebite research utilizing AI, including ML and deep learning, primarily focuses on snake classification/identification [15–18], this paper explores a joint application of ML and statistical design of experiment methodology. Although based on correlations, the relationships revealed by ML models can provide valuable insights when combined with statistical experiment design [13]. This innovative approach opens up new possibilities for understanding and combating SBE, instilling a sense of hope and optimism in the fight against this NTD.

This study employs advanced and innovative research methods, specifically a combination of statistical design of experiments and ML models. These include Ridge regression, Elastic Net, least absolute shrinkage and selection operator (LASSO), Generalized Regression Model with Pruned Forward Selection, and Forward Selection. These methods are applied under the null hypothesis that the WHO’s four strategic objectives for the control and prevention of SBE have the same priority among health workers, ensuring a comprehensive and rigorous approach to this study. The focus is exploring healthcare workers’ priorities regarding the WHO’s strategic objectives for controlling and preventing SBE. The goal is to provide a robust platform through rigorous research evidence to inform the development of a research agenda and relevant interventions or policies that prioritize local needs aligned with the WHO’s objectives for SBE control and prevention. This agenda has the potential to impact the fight against this NTD significantly. The perspectives of healthcare workers who treat and manage snakebite patients are crucial to fulfilling the goals of WHO. Healthcare workers are at the forefront of managing snakebite patients; their experiences and insights are invaluable to the health research agenda and development of relevant interventions or policies that meet local SBE needs.

There is a lack of research focusing on healthcare workers’ priorities regarding WHO’s strategic objectives for controlling and preventing SBE in various locations and countries. Nevertheless, a previous study in Ghana’s Ashanti and Upper West regions focused on healthcare workers’ rankings of the six key activities outlined in each of the WHO’s four strategic objectives using a qualitative assessment method [10]. The present study, conducted in the Kwahu Afram Plains North and South Districts of the Eastern Region of Ghana, where snakebites are a significant concern [19], aims to enhance the existing SBE literature by helping clinicians, researchers, and policymakers understand how we can leverage AI, including ML algorithms and MaxDiff statistical experiment designs to predict accurately the priorities that health care workers give to each of the WHO’s strategic objectives for SBE control and prevention. Furthermore, it seeks to provide evidence-based insights that can guide the development of a research agenda addressing local priority needs. This information, once integrated into local and regional healthcare policies and interventions, has the potential to significantly improve health outcomes and quality of life for snakebite patients in Ghana and other SSA regions.

## Methods

### Ethics

The study received ethical approval from the Ghana Health Service Ethics Review Committee (GHS-ERC073/04/24) and adhered to all ethical guidelines and regulations. After explaining the purpose of the study to all participants, written informed consent was obtained. Participants were informed that their participation was voluntary and that they had the option to choose whether to participate in the study or not.

### Study site

This cross-sectional study was conducted in the Eastern Region of Ghana, which comprises 9.5% of the country’s population [20]. It focused on two districts: Kwahu Afram Plains North District (KAPND) and Kwahu Afram Plains South District (KAPSD). These districts were selected due to the reported incidents of snake bites and related fatalities [19], a pressing public health issue. Both districts are situated in a savannah zone characterized by short, fire-resistant trees and varying grass heights [21,22]. Rainfall occurs from May to June and from September to October, with annual averages ranging between 1,150 mm and 1,650 mm. The dry season extends from November to late February, during which temperatures can reach highs of 36.6°C to 36.8°C and lows of 19.1°C to 20.1°C. The Afram and Volta rivers serve as the main drainage sources, supporting agriculture, fishing, and household activities throughout the year.

### Sample size and data collection

The minimum required number of respondents for this study was calculated to be 128, based on the sample size computation proposed by [23]. Therefore, the anticipated sample size of 137 respondents was deemed sufficient. We surveyed healthcare workers involved in the treatment and management of snakebite patients in the KAPND and KAPSD districts of the Eastern Region of Ghana from August to December 2024. Our goal was to identify their priorities regarding the WHO’s strategic objectives for controlling and preventing SBE. Before initiating the survey, we employed a multi-stage sampling technique and selected three communities—Donkorkrom, Tease, and Amankwakrom—as clusters for our sample. We then randomly selected 137 healthcare workers involved in treating and managing snakebite patients. This group included physician assistants, clinical officers, medical doctors, certificate/enrolled/general nurses, pharmacists, dispensing technicians, and community health nurses from three main healthcare facilities in each community. Various types of healthcare workers are involved in the management of SBE in Ghana [24]. We contacted the selected healthcare workers during working hours at their respective health facilities. Although some individuals declined to participate, we included only those healthcare workers who were willing to take part and provided written informed consent. Before starting the survey, we collected demographic data from participants and asked them some questions about their work experience and occupation/professional background.

### MaxDiff statistical experiment designs

In various fields such as healthcare, environmental sustainability, and consumer research, the MaxDiff approach is practically applied to elicit preferences for different goods and services [25–28]. In this method, respondents are asked to identify the best (most desired/preferred) and worst (least desired/preferred) options from a list of at least three attributes within a given choice scenario. The practical application of the MaxDiff method allows for the evaluation of the relative importance of these attributes across multiple scenarios. This approach, which requires greater cognitive effort and involvement than traditional rating scale tasks or interviews, was used in our study to enable respondents to provide a complete evaluation of the items presented to them [29]. We employed a statistical experiment block design [30] to randomly assign the WHO’s SBE strategic objectives into four choice scenarios of size three with three replicates. These choice scenarios were presented one at a time. Through an interviewer-administered questionnaire, participants were asked to indicate their most and least priority needs regarding the WHO’s strategic objectives. This information is intended to help inform the development of a research agenda and relevant interventions or policies that address local needs. Participants provided their responses using paper and pencil. To ensure clarity of the questionnaire, we conducted a pilot test with 20 healthcare workers. Participants were verbally informed about the WHO’s strategic objectives and encouraged to notify the interviewer if any part of the survey was unclear or challenging to answer. The positive feedback from participants regarding the clarity and ease of completion of the survey instills confidence in its design and implementation.

### Data preparation for model building

The data collected during our field survey showed no issues related to outliers or missing values. The study ensured that no data was absent by using an interviewer-administered questionnaire. Enumerators were present to conduct initial checks for any incomplete responses in the survey tools and encouraged respondents to finish any unfinished sections. To ensure the model’s generalization ability and mitigate the risk of overfitting, we carefully split the data using the hold-back validation method into training and validation sets, allocating 70% of the data for training and 30% for validation. It is important to note that the predictor variables related to the SBE strategic objectives were viewed as generic attributes, while the response variable, representing the subjective value assigned to each strategic objective, was considered continuous. We analyzed the final dataset using five ML models: Ridge Regression, Elastic Net, LASSO, Generalized Regression Model with Pruned Forward Selection, and Forward Selection. We selected these models as they consistently outperformed other candidates, such as the Support Vector Regression.

### Prediction based on five machine learning models

In this study, we analyzed the dataset using five ML models: Ridge Regression, Elastic Net, LASSO, Generalized Regression Model with Pruned Forward Selection, and Forward Selection. We assumed that the response vector $y=\;{\left(y_{1},...,\;y_{n}\right)}^{T}$ with n observations is a linear combination of p regressors $x_{1},...,x_{p}$ and an unknown parameter vector $\beta =\;{\left(\beta_{1},...,\beta_{p}\right)}^{T}$. The ML models can be formulated as follows.

**LASSO regression model:** This model that performs both variable selection and shrinkage determines the coefficient vector $\hat{\beta }$ satisfying


$$\begin{array}{c} \hat{\beta }\underset{\;\beta }{=\text{argmin}}\{\sum_{i=1}^{n}{\left(y_{i}-\sum_{j=1}^{p}x_{ij}\beta_{j}\right)}^{2}+\lambda \sum_{j=1}^{p}\left|\beta_{j}\right|\},\; \end{array}$$ (1)

where λ is regularization parameter. The LASSO imposes an L1-norm on the regression coefficients $\left|\beta_{j}\right|$, meaning the sum of the absolute value of the coefficients is restricted. This promotes the model’s sparsity by preventing overfitting by shrinking the model coefficients toward zero and performing variable selection.

**Ridge regression model:** This model adds an L2 penalty to the regression coefficients. This model yields more stable estimates by shrinking coefficients but does not select predictors, determines the coefficient vector $\hat{\beta }\;$ that satisfies


$$\begin{array}{c} \hat{\beta }\underset{\;\beta }{=\text{argmin}}\{\sum_{i=1}^{n}{\left(y_{i}-\sum_{j=1}^{p}x_{ij}\beta_{j}\right)}^{2}+\lambda \sum_{j=1}^{p}\beta_{j}^{2}\}. \end{array}$$ (2)

**Elastic Net model:** This model is a regularization technique that combines the LASSO and Ridge regression properties. It determines the coefficient vector $\hat{\beta }$ satisfying


$$\begin{array}{c} \hat{\beta }\underset{\;\beta }{=\text{argmin}}\{\sum_{i=1}^{n}{\left(y_{i}-\sum_{j=1}^{p}x_{ij}\beta_{j}\right)}^{2}+\lambda_{1}\sum_{j=1}^{p}\left|\beta_{j}\right|+\lambda_{2}\sum_{j=1}^{p}\beta_{j}^{2}\}, \end{array}$$ (3)

which depends on two regularizations $\lambda_{1},\;\lambda_{2}$. Elastic Net retains the benefits of this regularization and variable selection, as well as the grouping of correlated variables.

**Generalized Regression (Forward Selection) model** [13,31] improves model performance by removing insignificant variables. The process begins with no variables in the model, and at each step, the variable that contributes the most significant improvement to the model’s fit is added. The goal is to simplify the model while maintaining or enhancing its accuracy.

**Generalized Regression (Pruned Forward Selection) model** [13,31], which is a specific type of stepwise regression, is distinguished by its use of a pruning mechanism. This mechanism, which is not present in the forward selection, allows the method to enhance the process by incorporating a strategy to remove variables. After adding a new variable to the model, it evaluates whether any existing variables have become non-significant due to this addition. This iterative process of adding and removing variables continues until no significant improvement in the model can be achieved.

### Statistical analysis of data

To analyze the final dataset, we employed the five ML models and divided it using the hold-back validation method, maintaining a training-to-validation ratio of 70:30. To compare the models’ performance, we used several key metrics: the Akaike Information Criterion corrected (AICc), the Bayesian Information Criterion (BIC), the Root Average Squared Error (RASE), negative log-likelihood, and the total time taken to fit each model. These metrics help identify the best-performing model by evaluating the trade-offs between goodness of fit, model complexity, computational efficiency, and predictive accuracy. The practical implications of these findings are significant, as they can guide future model selection and application. Additionally, we assessed the significance of the WHO’s strategic objectives for controlling and preventing SBE at the 95% confidence interval (CIs). A utility estimate (UE) was considered statistically significant if the p-value was less than or equal to 0.001, 0.01, or 0.05 [28]. The sign of the utility estimate indicates whether the attribute has a positive or negative effect. All statistical analyses were conducted using JMP Pro (Version 17.0).

## Results

### Participant characteristics

A total of 137 participants took part in this study (see Table 1). Females comprised 60.6% of the participants. The median age of the participants was 34 years, with an interquartile range (IQR) of 30–37 years; 71.5% of the participants were at least 31 years old. Most participants (70.0%) were certified, enrolled, or general nurses and 43.8% had at least 6 years of work experience.

**Table 1:** Characteristics of study population.

Variable	Category	Respondents (*n* = 137)
Age (years) median (IQR)		34 (30–37)
Age (years)	18–30	39 (28.5%)
	≥ 31	98 (71.5%)
Gender	Males	54 (39.4%)
	Females	83 (60.6%)
Occupation	Physician assistants/Clinical officers/Medical doctors	16 (11.7%)
	Certificate/Enrolled/General nurses	96 (70.0%)
	Pharmacists/Dispensing technicians	12 (8.8%)
	Community health nurses	13 (9.5%)
Experience (years)	1–2	32 (23.4%)
	3–5	45 (32.8%)
	≥ 6	60 (43.8%)

### Model fit and overall performance

Table 2 compares the five candidate ML models: the Generalized Regression model with the LASSO, Pruned Forward Selection, Forward Selection, Elastic Net, and Ridge regularization. Among the models evaluated, the LASSO regression model stands out with the lowest AICc and BIC values of 2654.7183 and 2675.1545, respectively. These low AICc and BIC values indicate that the LASSO model offers the best-fit relative to the number of parameters used, effectively balancing model complexity and goodness of fit. The Elastic Net regression model closely follows, with AICc and BIC values of 2654.8070 and 2675.2433, respectively. Furthermore, the LASSO and Elastic Net models have slightly higher RASE values of 0.7448, suggesting they achieve slightly better prediction accuracy than other candidate models. However, it is important to note that the LASSO model (elapsed time = 1345 milliseconds) and the Elastic Net model (elapsed time = 1946 milliseconds) generally have the longest fit times, highlighting a trade-off between computational efficiency and goodness of fit. Regarding fit time, the Generalized Regression (Forward Selection) model is the most efficient, completing the fit in just 26 milliseconds. The Generalized Regression (Pruned Forward Selection) model follows closely with a fit time of 30 milliseconds. While achieving a lower goodness of fit, these models balance model complexity and goodness of fit more effectively than the Ridge regression model. However, they also exhibit the lowest predictive performance with a RASE of 0.7449, highlighting the trade-off between computational efficiency and predictive performance. This information provides a comprehensive understanding of the available options, helping to make informed decisions based on specific needs. While providing the highest AICc value of 2658.4903 and a BIC value of 2678.9266, the Ridge regression model stands out with its best predictive performance, with RASE of 0.7444, indicating a trade-off between goodness of fit and predictive performance. Furthermore, despite its longer fit time of 691 milliseconds, the Ridge regression model delivers superior predictive performance. This predictive performance instills confidence in its capabilities, making it a strong candidate model for applications where accuracy is paramount. Overall, the Ridge regression model is the best candidate, achieving higher computational efficiency and the lowest RASE/negative log-likelihood values. Its superior predictive performance or accuracy justifies the computational cost, making it the preferred choice for applications prioritizing predictive performance and computational efficiency.

**Table 2:** Model comparison.

Model	AICc	BIC	RASE	Elapsed time	Negative log-likelihood
LASSO	2654.7183	2675.1545	0.7448	1.3450	463.2057
Generalized Regression (Forward Selection)	2656.2267	2681.7638	0.7449	0.0260	463.2760
Generalized Regression (Pruned Forward Selection)	2656.2267	2681.7638	0.7449	0.0300	463.2760
Elastic Net	2654.8070	2675.2433	0.7448	1.9460	463.1931
Ridge	2658.4903	2678.9266	0.7444	0.6910	462.9046

AICc: Akaike Information Criterion corrected; BIC: Bayesian Information Criterion; RASE: Root Average Squared Error. The LASSO regression model has the lowest AICc and BIC values, indicating that it best fits the data. Regarding fit time, the Generalized Regression (Forward Selection) model is the fastest at 26 milliseconds, followed by the Pruned Forward Selection model at 30 milliseconds. Despite its longer fit time, Ridge regression has superior predictive performance, making it a strong candidate for applications prioritizing predictive performance and computational efficiency.

### Prediction profilers for each type of ML model

**Fig 1:**
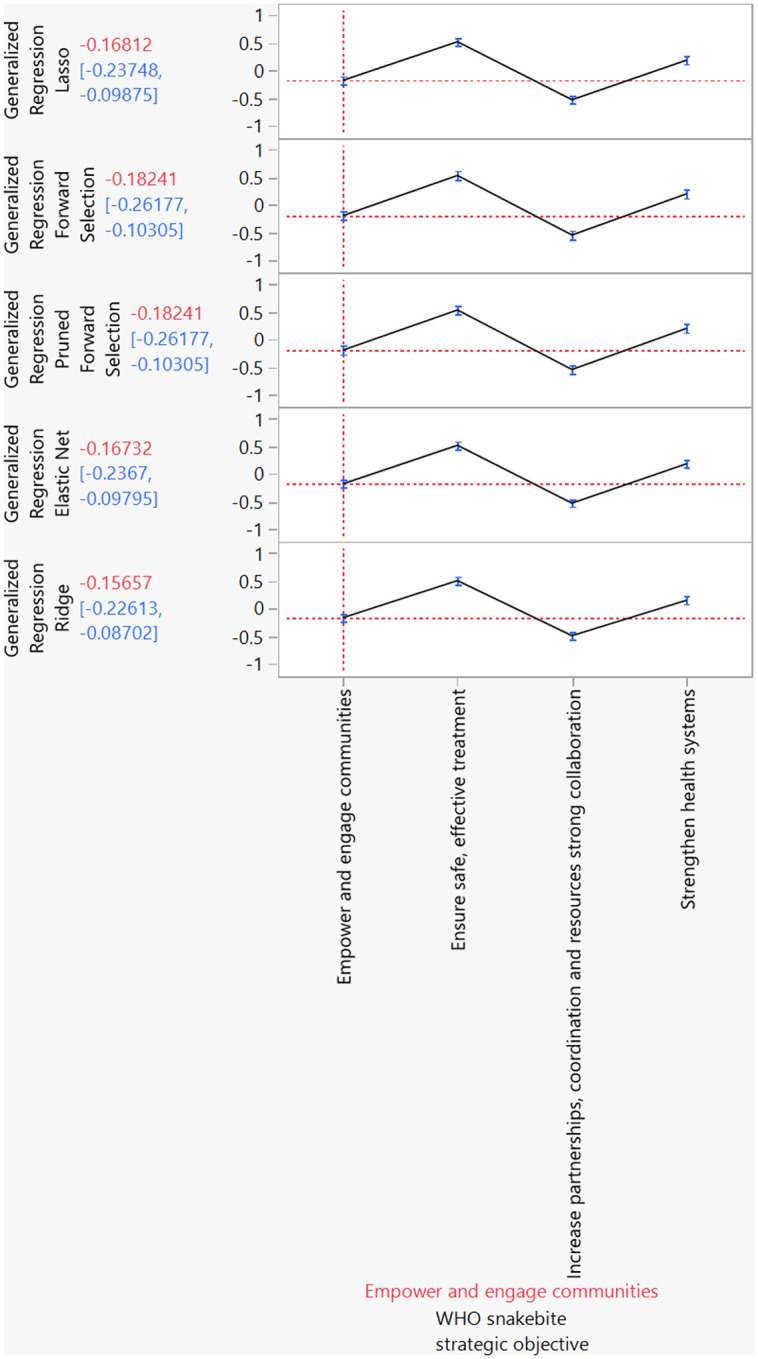
Prediction profiler for WHO SBE strategic objectives. While all the ML models consistently identified ‘Ensuring safe and effective treatment’, ‘Strengthening health systems’ and ‘Empowering and engaging communities’ as significant strategies for preventing and controlling SBE, ‘Increasing partnerships, coordination, and resources’ was least prioritized.

Fig 1 provides a visual representation of the utility profilers for each type of ML model. These profilers are instrumental in illustrating the importance assigned to the WHO’s strategic objectives for preventing and controlling SBE. The vertical red line in the utility profiler indicates the current value of the strategic objective ‘Empowering and engaging communities’. The horizontal red lines represent the current predicted values of each response variable based on this strategic objective. All the ML algorithms consistently identified: Ensuring safe and effective treatment’, ‘Strengthening health systems’, and ‘Empowering and engaging communities’ as significant strategies for preventing and controlling SBE. Despite its significance, ‘Increasing partnerships, coordination, and resources’ was least prioritized.

Magnitude of Scaled Parameter Estimates.

### Ridge regression model

We elected to present the results from the Ridge regression model because it demonstrated the best overall performance among all candidate models. Specifically, it achieved greater computational efficiency, the lowest RASE, and negative log-likelihood values. This computational efficiency is a practical benefit that makes it the preferred choice for applications prioritizing predictive performance and computational efficiency.

**Fig 2:**
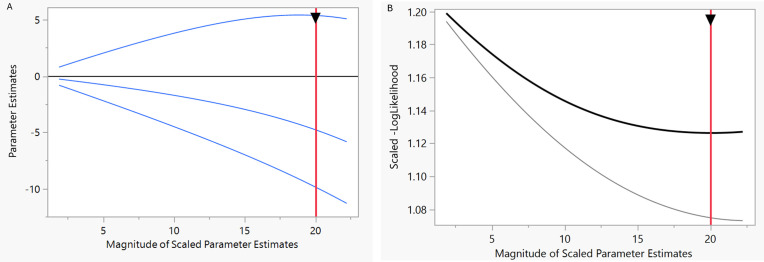
Solution path graph of the Ridge regression model. **(A)**: Characteristics of changes in attribute coefficients for WHO snakebite strategic objectives screening based on Ridge regression. **(B)**: The best model with the minimum scaled negative log-likelihood value for the validation set based on Ridge regression. The black and gray curves represent validation and training components, respectively.

Fig 2 presents the solution path graph of the Ridge regression model, a crucial tool for understanding the model and its fitting process. This graph, which shows plots of the parameter estimates and scaled negative log-likelihood, is key to understanding the model. The vertical red line indicates the parameter estimate values at the optimal shrinkage (Lambda penalty = 0.1092), as determined by the holdback sample defined by the cross-validation criterion. The Ridge regression model includes four attributes related to the WHO’s strategic objectives for preventing and controlling SBE. The changes in the coefficients of these strategies are illustrated in Fig 2A. The best model, which has the minimum scaled negative log-likelihood value of 462.9046 for the validation set, is shown in Fig 2B with black and gray curves representing the validation and training, respectively. Finally, three strategic objectives, as shown in Table 3, were screened: ‘Ensuring safe and effective treatment’, ‘Empowering and engaging communities’, and ‘Increasing partnerships, coordination, and resources’.

**Table 3:** Ridge regression model results.

Attribute	Utility estimate	SE	Wald ChiSquare	Prob > ChiSquare	Lower 95%	Upper 95%
Empowering and engaging communities	-0.3148	0.0579	29.5483	<.0001*	-0.4283	-0.2012
Ensuring safe and effective treatment	0.3552	0.0574	38.2838	<.0001*	0.2427	0.4678
Increasing partnerships, coordination and resources	-0.6391	0.0576	122.7556	<.0001*	-0.7522	-0.5261
Strengthening health systems^a^						
**Model Fit Statistics**						
Wald Chi-Square	307.4839					
AICc	2658.4903					
BIC	2678.9266					
RASE	0.7444					
Negative Log-Likelihood	462.9046					
DF	3					
P-value	0.0001*					

^a^Reference attribute; AICc: Akaike Information Criterion corrected; BIC: Bayesian Information Criterion; RASE: Root Average; SE: Squared Error; DF: Degree of freedom. All the WHO strategic objectives are statistically significant, as the 95% confidence intervals do not include zero.

Relative to the reference attribute, the model identifies significant strategic objectives for preventing and controlling SBE (Table 3). These include: ‘Empowering and engaging communities’ (UE = -0.3148, 95% CI: -0.4283, -0.2012), ‘Ensuring safe and effective treatment’ (UE = 0.3552, 95% CI: 0.2427, 0.4678) and ‘Increasing partnerships, coordination, and resources’ (UE = -0.6391, 95% CI: -0.7522, -0.5261). Among these, ‘Ensuring safe and effective treatment’ is the top priority. This suggests a strong need to prioritize this strategy when designing policies and developing a research agenda. Despite their significance, participants tend to trade off or least prioritize the objectives of ‘Empowering and engaging communities’, followed by ‘Increasing partnerships, coordination, and resources’.

### Multiple comparisons of WHO SBE strategic objectives

**Fig 3:**
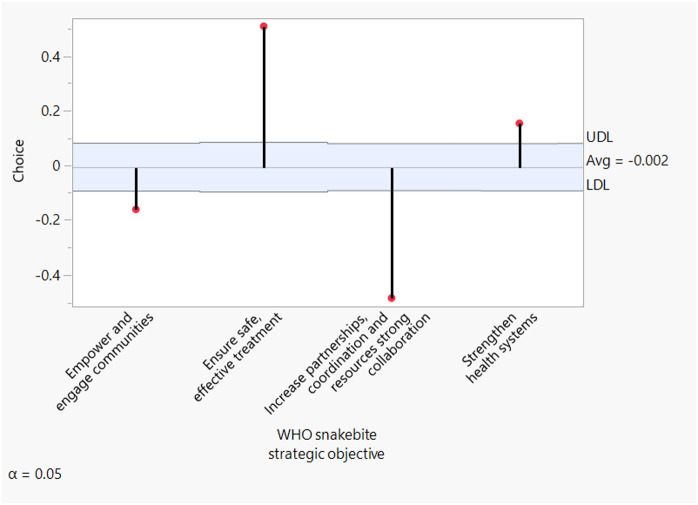
Overall average decision chart. The strategic objective ‘Ensuring safe and effective treatment’ has the highest average priority, followed by the objectives ‘Strengthening health systems and ‘Empowering and engaging communities. The objective ‘Increasing partnerships, coordination, and resources’ has the lowest average priority.

Fig 3 illustrates the multiple comparisons of the WHO snakebite strategic objectives alongside the overall average decision chart. It presents the group average, the upper decision limit (UDL), and the lower decision limit (LDL). A snakebite strategic objective is considered statistically significant if it exceeds the decision limits. The averages for all the strategic objectives differ significantly from the overall average. Specifically, the objectives ‘Ensuring safe and effective treatment’ (lower limit = -0.0929, upper limit = 0.0881, difference from overall average = 0.5135, p-value = 0.0001) and ‘Strengthening health systems’ (lower limit = -0.0892, upper limit = 0.0845, difference from overall average = 0.1582, p-value = 0.0001) have higher averages. In contrast, the objective ‘Empowering and engaging communities’ (lower limit = -0.0899, upper limit = 0.0852, difference from overall average = -0.1565, p-value = 0.0001) has lower average, followed by ‘Increasing partnerships, coordination, and resources’ (lower limit = -0.0883, upper limit = 0.0835, difference from overall average = -0.4809, p-value = 0.0001).

## Discussion

This study utilized advanced and innovative research methods, notably a combination of a MaxDiff statistical experiment design and machine learning models. Our primary focus was to explore the priorities of healthcare workers in a particular region of Ghana regarding the WHO’s strategic objectives for controlling and preventing SBE. The goal was to establish a robust foundation through rigorous research evidence to guide the development of a research agenda and relevant interventions or policies that cater to local needs while aligning with the WHO’s SBE objectives. Our findings hold significant relevance to clinicians, researchers, and policymakers. Identifying country and region-level needs is crucial for devising effective strategies that address local concerns and support the WHO’s goal of reducing the global burden of SBE by 50% by 2030.

The Ridge regression model emerged as the best candidate among the ML models, a reassuring result regarding the reliability of our research. Its superior predictive performance/accuracy justifies the computational cost, making it the preferred choice for applications prioritizing prediction and computational efficiency. Among the strategic objectives of the WHO SBE, ‘Ensuring safe and effective treatment’ was the highest priority. This is followed by ‘Strengthening health systems’, ‘Empowering and engaging communities’, and ‘Increasing partnerships, coordination, and resources’. Thus, our findings do not support the null hypothesis that the WHO’s four strategic objectives for the control and prevention of SBE have the same priority among these health workers. The order of prioritization of these objectives underscores their significant impact on local initiatives. Therefore, these strategies should be prioritized when developing local policies, relevant interventions, and research agendas. In SSA, the primary objective of snakebite interventions should be improving care through multiple actions [32].

Our findings underscored that healthcare workers perceived ‘Ensuring safe and effective treatment’ as the top local priority among the WHO’s strategic objectives. This highlights the urgent need to prioritize this objective when designing local policies and interventions and developing a research agenda. The emphasis on this aspect of the WHO strategy can be understood in the light of the deficit in the availability and accessibility of safe and effective antivenoms in SSA, whereby a combination of lack of antivenoms and the presence of antivenoms of dubious efficacy and safety represent a significant problem [33,34]. This has prompted renewed efforts to improve the treatment of SBE, such as the risk-assessment program of the WHO to evaluate the suitability of products being distributed in SSA, the production of antivenoms by manufacturers outside SSA [35,36], efforts to establish new facilities for antivenom production in SSA [32], guidelines for the training of healthcare workers on the management of SBE [6], and multidisciplinary research initiatives in the region [11]. Addressing this local priority need is crucial for effectively reducing the global burden of SBE by 2030. As efforts to achieve the objectives of UHC2030 (https://www.uhc2030.org/) gain momentum, immediate local action plans on SBE, such as those initiated by India [37], and advocacy for various advanced research needs are crucial. These include investigating the incidence and mortality of the disease [2], conducting cost-effectiveness analyses of antivenoms [38], basic studies on venom composition and mechanisms of action [39,40], assessing antivenom preclinical efficacy [41], carrying out well-designed randomized controlled trials on antivenom efficacy [42], explore new therapies [43], and improve diagnostics to improve the management of SBE [44].

Our results showed that healthcare workers also prioritized the objective of ‘Strengthening health systems’ at the local or community level as vital for supporting the WHO’s overarching goal of reducing the global burden of SBE [9]. SBE is an acute emergency that necessitates health systems capable of delivering timely services [32]. Therefore, integrating practical and effective approaches to treating and managing SBE into national health policy and health systems is essential. Previous studies have highlighted the need to address challenges within health systems, including the training of health professionals and building the capacity of community health centers closest to areas where snakebites are common to reduce delays in managing SBE and decrease both the mortality rate and the incidence of complications, such as permanent disabilities [32,45–47]. This is particularly crucial since many snakebite cases occur in rural areas, where antivenoms are often unavailable [32,48,49], making them largely inaccessible to those who need them most [47]. It is imperative to develop standardized local treatment protocols, including pre-hospital care, and deploy them in rural health facilities [10,50].

This study also highlighted ‘Empowering and engaging communities’ as a significant strategy when developing local policies, relevant interventions, and research agendas to reduce or mitigate deaths, disabilities, and socioeconomic impacts caused by SBE. It is essential to empower and engage affected and remote/rural communities by providing them with accurate information, particularly through education and training on the prevention and control of SBE [51,52]. There is an urgent need to design and develop resources for community health education programs and to implement them in Ghana and the sub-region. These programs should raise awareness about SBE among rural communities, healthcare professionals, and health authorities. Various educational tools—such as information leaflets, posters, pocket guides, learning materials for healthcare professionals, and short or long video documentaries in local languages—can effectively engage target communities. These materials should be distributed through direct community gatherings, events, and mass communication channels, including traditional and social media. Such efforts can improve awareness, treatment-seeking behavior, and clinical practices [53,54]. However, implementing these community engagement activities requires the commitment of individuals, local government, health authorities such as the Ministry of Health/Ghana Health Service, and personnel at Community-Based Health and Planning Services compounds. Having trained personnel who reside in the same communities can enhance trust and compliance with the proposed programs [52], and it can also lead to changes in the treatment-seeking behavior and lifestyle of rural communities to prevent SBE incidents. Considering the local knowledge, attitudes, and health beliefs regarding SBE at the community level is crucial [54]. This ensures that the community education interventions are targeted to meet their needs and are culturally appropriate [54]. While community education should focus on the prevention of SBE, it should also encompass appropriate first-aid management. Additionally, community services must be established to support victims who may suffer from long-term physical disabilities as well as psychological issues such as depression and post-traumatic stress disorder [55].

Although the objective of ‘Increasing partnerships, coordination, and resources’ is a significant component of the strategy, it was the least priority among health workers in our study. Nonetheless, the rationale behind the WHO strategy is based on an integrated approach to tackling SBE. Therefore, the overall efforts to set agendas and develop local policies and interventions to address SBE must strike a balance among the four WHO objectives. In light of recent global funding cuts [56], which have disrupted health services—including the supply of medicines and other health products—local governments must prioritize and address the significant economic burden of SBE. They should take necessary measures to revise budgets, optimize costs, and strengthen partnerships and fundraising efforts to support investments in equipment and staff and fund antivenoms, as there is currently no dedicated financial support for snakebite therapies to enhance accessibility to proper antivenoms and symptomatic treatments [32]. Local initiatives, such as the newly established Ghana National Research Fund, aimed to promote research and innovation nationwide [57], should fund high-impact research projects, such as those investigating new therapies and comprehensive population-based SBE surveys, and support capacity-building and training programs for health personnel. Coordinating donations from international organizations, private companies, and NGOs is therefore essential.

This study has limitations. The results of this work may have broader implications for other regions, as the probability sampling technique and validation approach used in the ML models allow for generalizability and scalability. However, due to a lack of funding, the study was limited to two districts and healthcare workers. Therefore, establishing funding schemes for country-level researchers to conduct comprehensive population-based surveys that incorporate the perspectives of other stakeholders, such as community members, policymakers, and local snakebite researchers, is crucial and should be a top priority for the WHO and regional/global funders. It is important to develop further research to determine if there are significant differences in priorities regarding the WHO’s SBE strategic objectives in Ghana and the sub-region. The interview-administered survey may introduce interviewer bias, despite not allowing real-time observation of responses and permitting self-administration of the questionnaire upon request. Additionally, this study reflects the perspectives of healthcare workers, potentially biasing the findings toward their professional needs. Future research should also consider the perspectives of other stakeholders, such as community members, policymakers, and local snakebite researchers when setting research agendas related to SBE and developing relevant local plans or policies. This collaborative approach is crucial to achieving the WHO objectives and broadening the landscape of sectors involved [58].

In conclusion, we have demonstrated that generating firm conclusions about local priority needs about the WHO’s SBE strategic objectives can benefit from advanced statistical design of experiments method and artificial intelligence/machine learning models that are predictive, interpretable, and computationally efficient. This understanding will establish a solid foundation for discussions on setting the research agenda for SBE and prioritizing relevant local policies. Our findings offer valuable insights into local policymaking and intervention strategies and into shaping research agendas in Ghana. There is an urgent need for a local action plan that prioritizes ‘Ensuring safe and effective treatment’ at the community level, followed by ‘Strengthening health systems’, ‘Empowering and engaging communities’, and ‘Increasing partnerships, coordination, and resources’. Prioritizing these strategies in Ghana is vital for supporting the WHO’s goal of reducing the global burden of SBE by 50% by 2030. However, the success of these strategies hinges on the active involvement of the Ministry of Health and the Ghana Health Service in their implementation at the local level and within the health system.
